# Access to healthcare services and confidence in healthcare professionals’ management of malaria: the views of Francophone sub-Saharan African Immigrants living in western Canada

**DOI:** 10.1186/s12889-023-17266-3

**Published:** 2023-12-08

**Authors:** Rémi Vincent, Kongnon Sangué Coulibaly, Ali Ahmed, Youssef Ahmed, Taylor A. Hanna, Srilata Ravi, Michael T. Hawkes, Sedami Gnidehou

**Affiliations:** 1 Faculté Saint-Jean, University of Alberta, Edmonton, AB Canada; 2 Faculty of Science, University of Alberta, Edmonton, AB Canada; 3 Department of Pediatrics, Faculty of Medicine, University of Alberta, Edmonton, AB Canada; 4 Department of Medical Microbiology and Immunology, Faculty of Medicine, University of Alberta, Edmonton, AB Canada; 5 School of Public Health, University of Alberta, Edmonton, AB Canada

**Keywords:** French speaking-immigrant, Sub-Saharan Africa, Access to Care, Health professional, Quantitative, Malaria

## Abstract

**Background:**

There is a paucity of knowledge about the healthcare attitudes and practices of French-speaking immigrants originating from Sub-Saharan Africa (FISSA) living in minority settings. The purpose of this study was to characterize FISSA healthcare experiences and confidence in the malaria-related knowledge of health professionals in Edmonton.

**Methods:**

A structured survey was used to examine a cohort of 382 FISSA (48% female; 52% male) living in Edmonton. FISSA general healthcare attitudes, experiences and satisfaction with the Canadian healthcare system were studied. Healthcare Competency Perception (HCP) was characterized by using an index score. Statistical analyses were performed to evaluate the impact of healthcare experiences and other outcomes.

**Results:**

Intriguingly, while only 42% of FISSA had a French-speaking family physician, 83% (197/238) of those who had received health care services in Alberta found that access to medical treatment was easy, and 77% (188/243) were satisfied with received care. Although 70% (171/243) of FISSA did not receive services in French, 82% (199/243) surprisingly reported having good levels of comprehension during their visits. Satisfaction with care was associated with having a family physician (*p* = 0.018) and having health insurance (*p* = 0.041). Nevertheless, confidence in the healthcare system’s ability to treat malaria effectively was significantly lower, with only 39% (148/382) receiving a positive score on the HCP index.

**Conclusion:**

This study provides an important insight into FISSA experience with and perception of the Alberta’s healthcare system.

**Supplementary Information:**

The online version contains supplementary material available at 10.1186/s12889-023-17266-3.

## Background

The ability to access healthcare services is an integral part of well-being and represents a key indicator of immigrant integration in their host countries [1]. Factors that influence immigrants’ use of health services include length of time since immigration, knowledge of the healthcare system, differences in treatment preferences, perceived service quality, economic factors like income and insurance, as well as sociodemographic factors [2].

In Canada, immigrants make up approximately 22% of the population [3]. Despite the “healthy immigrant effect”, a phenomenon in which immigrants tend to be healthier than the general Canadian population upon arrival to Canada, many immigrants struggle to navigate the new healthcare system and face barriers in accessing healthcare services [4]. Hurdles include language barriers, cultural differences, shortage of healthcare providers, need of transport services to access care resources, lack of social support and medication costs [4, 5]. Challenges also include immigrant’s level of confidence in the Canadian healthcare system and its ability to meet their unique needs. Such confidence was influenced by ethnicity and English proficiency [6]. Here, confidence refers to individuals believing in the quality and effectiveness of medical care to influence choices and decisions [7].

Most of our knowledge of immigrants’ experiences regarding access to healthcare in Canada stems from research that focused mainly on specific ethnic groups, particularly South Asians, Chinese [4, 8] and English-speaking Black Canadians [9]. Far less is known about the experiences of Francophone immigrants originating from sub-Saharan Africa (FISSA) living in linguistic minority settings. When available, healthcare related information characterizing this unique population is often combined with the analyses of either African immigrants [10], Francophone populations [11, 12], Canadian immigrants at large [4], or an undifferentiated pool of Black Canadians [13, 14].

Alberta is a predominantly English-speaking province located in western Canada. From 2004 to 2014, Alberta’s economy saw the strongest economic growth rate of all Canadian provinces [15], encouraging immigrants such as FISSA to settle [16]. The 2016 Census of Canada estimated 3765 FISSA permanent residents admitted into this province from 2000 to 2019 [3]. With the increasing number of FISSA living in Alberta, FISSA represent a significant proportion of Alberta’s French-speaking population [16].

Several lines of evidence have highlighted a strong association between French speakers residing in predominantly English-speaking communities and a variety of difficulties with healthcare outside Quebec, including decreased confidence in services received and patients’ ability to successfully receive care [17, 18]. For example, language was shown to hinder Francophone Africans from being tested for HIV in Canada [19]. While it has been reported that both immigrant and non-immigrant francophones experience similar difficulties when attempting to find family doctors [12], we consider that factors such as differing social and cultural practices may contribute to additional risks for populations like FISSA when it comes to diseases that are endemic in their country of origin. This is emphasized by our previous study [20] which demonstrated that despite originating from SSA, where approximately 95% of annual malaria cases and deaths are reported, only 52% of FISSA travellers had booked pre-travel medical consultations before travelling to malaria-endemic settings. In contrast, travellers of Canadian origin have been shown to systematically visit doctors before travelling to these countries [21]. Furthermore, globalization has led to increased physician exposure to non-endemic diseases such as malaria in Canada. This puts a burden on medical practitioners, as previous studies have found that host countries’ physicians show a low level of comfort in managing tropical diseases due to minimal knowledge, training and experience [22, 23]. This may create some concerns among immigrants regarding physicians’ ability to respond to their medical concerns, including the management of tropical diseases like malaria.

Collectively these findings warrant further investigation to determine FISSA health-related views and experiences in accessing healthcare services, and to examine FISSA’s confidence in healthcare providers’ knowledge and management of malaria, a disease that is non-endemic in Canada and frequently perceived as unfamiliar to Canadian health providers [1].

In this study, we describe the first cross-sectional study conducted in Edmonton to determine FISSA's view on healthcare professionals and their experiences with healthcare services. Our primary objective was to characterize FISSA general health attitudes by examining health practices since arrival to Canada, experiences and resulting satisfaction with the healthcare system. FISSA are particularly at risk of contracting imported malaria (IM) or travel-associated malaria. While several barriers contributing to lower confidence in health professionals’ ability to effectively prevent and treat malaria could lower this population’s use of related services and result in increased susceptibility to IM infections, we characterized FISSA confidence levels using a Healthcare Competency Perception (HCP) index score.

## Methods

Most methods used have been described in detail previously [20].

### Study settings and participants

At the time of data collection (2018–2019), the population of Edmonton’s metropolitan region was estimated to be 1,321,426 and to include 27,100 individuals defining themselves as Francophone [24]. A total of 500 eligible participants over the age of 18 were invited to complete a survey with the conditions that French was their first language, that they identified themselves as first or second- generation FISSA and that they were living in the Edmonton Metropolitan Region. Participants were informed through posters and settlement’s counselors [20]. FISSA were recruited in several areas in the Edmonton Metropolitan region and suburbs between September 2018 and March 2019 as previously described in [20]. Exclusions were made if participants reported never having heard of malaria or if they had completed the survey despite not meeting all aforementioned criteria. Cross-sectional data were obtained from 409 FISSA.

### Survey questionnaire

Details related to the survey pre-testing, validation and implementation were described elsewhere [20]. In brief, an online and paper-based survey questionnaire written in secondary-level French was used to collect participants' socio-demographic information and to assess healthcare-related attitudes and preferences such as changes to health practices since arrival in Canada, preferred types of care and most important factors when looking for healthcare. Questions concerning overall experience with the Albertan healthcare system and their perception of healthcare professionals’ competency regarding malaria were also included in the survey. The online version of the questionnaire was hosted by the KoBo Toolbox application (Version 2.019.52). Data collected from all paper submissions were manually entered into the KoBo Toolbox database. Both the manually and electronical entries were verified. A Microsoft Excel spreadsheet containing data from all 409 participants was generated from a KoBo Toolbox (Version 2.019.52) database. As previously described, data from 382 respondents were used for analysis.

## Measures

### Accessing FISSA general healthcare attitudes

Key questions focusing on the type of preferred care, factors FISSA consider when seeking care as well as explanation for their choices were asked. For questions that allowed participants to give multiple answers (i.e., if a participant mentioned their preferred type of care as being both “biomedicine” AND “traditional medication”), multiple answers were recorded, and the total number of answers provided (*n* > 382) surpassed that of the participants (*n* = 382). This was done to ensure proper representation of participants’ answers during analysis.

### Satisfaction with care received

Only the 243 participants who mentioned having received care in Alberta were considered to reflect the healthcare experience. FISSA satisfaction with care received was assessed on a Likert scale ranging from 1–5 (strongly disagree – disagree – neutral – agree – strongly agree). Questions included demographic information and items related to perceptions of healthcare experience.

### Estimation of healthcare competency perception (HCP)

Three questions related to FISSA perception of Canadian doctors’ malaria knowledge were included. The questions involved FISSA perception of Canadian physicians’ ability to recognize malaria and hospitalize and do testing only when necessary. The answers were assessed on a Likert scale ranging from 1–5, like the satisfaction with care received. The answers to the three questions were combined in a latent factor analysis to build the HCP index. Further details of the survey and analyses are provided in Supplementary file 1.

### Statistical analysis

The R statistical environment was used [25]. Descriptive analysis included percentages and frequencies. To examine associations between variables/factors, Odds Ratio (OR) and Fisher’s exact test were performed. A latent variable [26] was created, using a latent factor analysis combining the answers to the relevant questions, and applied to assess FISSA’s perception of and confidence in the Healthcare practitioners' competency regarding malaria care. Further details are provided in Supplementary file 1. Confidence interval and statistical significance were set at 95% (*p* ≤ 0.05) respectively.

## Results

### Study cohort

Table 1 presents socio-demographic characteristics of the 382 participants regarding relevant healthcare accessibility criteria: Having a family physician, having a French speaking family physician, and having health insurance. Eighty-nine percent of FISSA reported having health insurance. As previously demonstrated, 84% (321/382) of FISSA stated French as the language in which they were most at ease, with 77% (248/321) having a family doctor [20]. However, of those reporting French as their language of preference, only 46% (114/248) had a French speaking family physician. Participants who had lived in Canada for longer were more likely to have a family physician and/or health insurance. While 91% (259/283) of first-generation FISSA who had lived in Canada for more than three years indicated having health insurance, this percentage dropped to 80% (66/82) for first-generation FISSA who had lived in Canada for shorter periods of time (*p* = 0.021). Additionally, 81% (228/283) of established first-generation FISSA (more than 3 years) had a family physician compared to 65% (53/82) of recent immigrants (3 years or less) (*p* = 0.01).

**Table 1:** Sociodemographic characteristics of participants and healthcare accessibility

**Parameter**	**Has a family physician**	**Physician is French speaking**^(a)^	**Has health insurance**	**Total number of participants**
	n (%)	n (%)	n (%)	
**Gender**				
Female	143 (78)	65 (45)	159 (87)	183
Male	151 (76)	57 (38)	179 (90)	198
Other	1 (100)	0 (0)	1 (100)	1
**Age (31.3 ± 11.25)**^(b)^				
18 – 29	113 (66)	45 (40)	154 (90)	171
30—44	119 (84)	46 (39)	126 (89)	142
45—59	55 (92)	28 (51)	52 (87)	60
60 +	8 (89)	4 (50)	7 (78)	9
**Family with children (≤ 5 y/o)**				
Yes	90 (97)	91 (44)	83 (89)	93
No	205 (71)	32 (36)	256 (89)	289
**Place of birth**				
Sub-Saharan Africa^(c)(d)^	283 (78)	120 (42)	325 (89)	365
Canada^(e)^	6 (86)	0 (0)	6 (86)	7
Other^(d)^	6 (60)	3 (50)	8 (80)	10
**Years since arrival in Canada**^(f)^				
3 or less	53 (65)	25 (47)	65 (79)	82
More than 3	233 (81)	97 (42)	264 (91)	289
**Education**				
No formal education	5 (56)	3 (60)	8 (89)	9
Primary	11 (100)	2 (18)	11 (100)	11
Secondary	50 (74)	18 (36)	62 (91)	68
Higher	227 (78)	100 (44)	255 (88)	291
No answer	2 (67)	0 (0)	3 (100)	3
**Language**^(g)^				
French	248 (77)	114 (46)	285 (89)	321
English	27 (77)	2 (7)	31 (89)	35
French and English	15 (88)	6 (40)	15 (88)	17
Other^(h)^	5 (56)	1 (20)	8 (89)	9

^a^*n* = 295. Only includes those who have a family physician

^b^Range: 18–74 years

^c^List of countries in sub-Saharan Africa includes Benin, Burkina Faso, Burundi, Cameroun, Central African Republic, Ivory Coast, DRC, Gabon, Ghana, Guinea, Mali, Mauritania, Niger, Rwanda, Senegal, Tchad and Togo

^d^Considered first-generation in study. Defined as people who were born outside of Canada

^e^Considered second-generation in study. Defined as People who were born in Canada and have at least one parent born outside of Canada

^f^Only includes the first-generation participants who answered the question

^g^Language in which respondents are most at ease

^h^“Other” refers to people who were at ease in a language other than English or French

### FISSA Health attitudes and behaviour changes

FISSA’s general healthcare attitudes and valuable factors when looking for health services are presented in Table 2. While biomedicine was the preferred type of care (77%), traditional and homeopathic treatments were also commonly listed (16% and 9% respectively). There was heterogeneity regarding important factors when looking for healthcare as well as the reasons given for those factors (Table 2). Factors were divided into three main categories related to care, access, and interpersonal skills. FISSA placed the most value on care factors including competency (18%) and quality of care (14%). They considered proximity (12%) and language (12%) to be of equal importance. Factors like welcoming environment (7%), short wait times (7%), and cultural competency (3%) were also mentioned. An insight into FISSA health behavior was described in Table 3. Only 39% (148/376) of FISSA indicated having changed their health practices since arrival. The most cited improvements were more frequent checkups (35%, 52/148) and improving sanitary practices (32%, 47/148). Curiously, there was a considerable number of participants (43%) who indicated not having made any changes. Of those, 53% (86/162) felt no need to make such changes. No significant associations were found between having changed health practices and having health insurance or having lived in Canada for more than 3 years.

**Table 2:** FISSA’s general healthcare attitudes

Parameter	Frequency (*n* = 382)	Percentage (%)
**Type of care preferred**^a^		
Biomedicine	295	77
Traditional medication	60	16
Homeopathic medication	35	9
No preference	33	9
Not a fan of medication	2	1
**Most important factors when looking for healthcare**^(a)(b)^		
***Care factors***	*135*	*39*
Competency	64	18
Quality of care	47	14
Efficiency	13	4
Professionalism	11	3
***Access factors***	*123*	*36*
Proximity	43	12
Accessibility	26	8
Short wait times	24	7
Price	19	5
Availability	11	3
***Interpersonal factors***	*103*	*30*
Language	42	12
Welcoming environment/customer service	25	7
Communication/listening skills	21	6
Cultural competency	9	3
Confidence	6	2
Recommendations / online ratings	11	3
Other ^(c)^	114	33
**Reasons given for the importance of those factors**^(a)(d)^		
Improves care/satisfaction/health	90	28
Saves time/improves access	62	19
Helps describe/understand	46	14
Prevention/reduces risk of bad outcomes	33	10
Comfort/trust	20	6
Other^(e)^	151	57

^a^ =multiple answers possible—participants who gave two or more answers are included in all answers’ frequencies

^b^*n* =346 participants who answered. Excludes 36 participants who did not respond

^c^“Other” includes research, prevention/no side effects, healthy lifestyle/wellbeing, I don’t know, etc

^d^*n* =320 participants who answered. Excludes 62 participants who did not respond

^e^“Other”includes finances, crucial for research, important, I don’t know, no response, no reason, etc

**Table 3:** FISSA general healthcare practices since arrival in Canada

**Parameter**	**Frequency (*n* = 376)**^(e)^	**Percentage (%)**
**Have changed health practices**^(a)^		
***Yes***^(b)^	148	38
More frequent checkups	52	36
Sanitary (personal hygiene, clean environment, food storage)	47	32
Diet and exercise	15	10
Follow medical advice/prevention/cautious	15	10
Access to more products/services	9	6
Other^(c)^	36	25
***No***^(d)^	158	43
No need	86	52
Still have the same family doctor	8	5
Malaria is not present in Canada	4	2
Still have the same health issues	3	2
Recently immigrated to Canada	2	1
Other^(c)^	49	30
***Do not know***	70	19

^a^ = multiple answers possible—participants who gave two or more reasons are included in all answers’ frequencies

^b^*n* = 148 participants who changed health practices since arrival in Canada

^c^“Other” includes participants who mentioned other factors, did not respond, or gave no reason

^d^*n* = 158 participants who did not change health practices since arrival in Canada

^e^*n* = 376 participants—excludes participants born in Canada

### FISSA’s experience with the Alberta health care system

Sixty-four percent (243/382) of FISSA have reported having received health care services in Alberta (Tables 4 and 5). Of those, 83% found that access to medical treatment was easy, and 77% were satisfied with the care they received (Table 4). Alongside, 71% of parents were also satisfied with the care their child received. Having a family physician and satisfaction with care were associated. Eighty percent of FISSA who reported having a family physician were satisfied with their care compared to 61% of those who did not have one (*p* = 0.018). Furthermore, having health insurance was also positively associated with satisfaction levels. While 80% of FISSA who reported having health insurance were satisfied with their care, only 47% of participants without insurance were satisfied (*p* = 0.004). Language of communication and comprehension are important parameters to consider when assessing satisfaction with received care. Even though 70% (171/243) did not receive services in French, 82% still mentioned having good comprehension during their visits (Table 4). For patients who indicated being more comfortable in French, low comprehension levels (≤ 5/10) were only reported by 3.7% (2/54) if French care had been received. For those who had received care in English however, 12% (15/127) had reported low comprehension.

**Table 4:** FISSA’s experience with the Albertan healthcare services

Parameter	Frequency (*n* = 382)	Percentage (%)
**Number of visits per year**		
More than two	155	41
Two or less	150	39
Other^(a)^	77	20
**Have received healthcare services in Alberta**	243	64
**Did not received healthcare service in French**^(b)^	171	70
**Felt access to medical treatment was easy**^(c)^		
Yes	197	83
No	33	14
Unsure	8	3
**Self-rated comprehension during visits (1–10 scale)**^(b)^		
Good (> 5)	199	82
Low (≤ 5)	20	8
Other^(d)^	24	10
**Satisfied with care**^(b)^		
Yes	188	77
Neutral	44	18
No	11	5
**Satisfied with care of children**^(e)^		
Yes	172	71
Neutral	59	24
No	12	5

^a^“Other” includes participants who were unclear, answered “N/A” or did not answer

^b^*n* = 243: Only includes participants who answered “Yes” to having received care in Alberta

^c^*n* = 238: Includes participants who answered “Yes” to having received medical treatment in Alberta and answered the treatment accessibility question

^d^“Other” includes participants who were unclear or did not answer

^e^*n* = 243: Only includes participants who indicated having children

**Table 5:** FISSA satisfaction with care received and sociodemographic parameters (*n* = 243)

Parameter	I am satisfied with my care			
	Strongly Agree/ Agree	Neutral	Disagree/Strongly Disagree	*P* value
**Gender**				
Female	91 (77)	21 (18)	6 (5)	0.97
Male	97 (78)	22 (18)	5 (4)	
**Age (Year)**				
18–44	146 (76)	36 (19)	9 (5)	0.91
45–74	42 (81)	8 (15)	2 (4)	
**Years since arrival in Canada**^(b)^				
3 or less	31 (72)	12 (28)	0 (0)	0.06
More than 3	154 (79)	30 (15)	11 (6)	
**Comprehension language**^(c)^				
French	160 (77)	37 (18)	10 (5)	0.83
English	16 (73)	5 (23)	1 (5)	
**Family physician**				
Yes	165 (80)	31 (15)	9 (4)	***p* < 0.05**
No	23 (61)	13 (34)	2 (5)	
**French speaking family physician**^(d)^				
Yes	69 (83)	12 (14)	2 (2)	0.57
No	95 (79)	18 (15)	7 (6)	
**Health Insurance**				
Yes	179 (80)	36 (16)	9 (4)	***p* < 0.01**
No	9 (47)	8 (42)	2 (11)	
**Self-rated comprehension**^(e)^				
Low (≤ 5)	15 (75)	4 (20)	1 (5)	0.91
Good (> 5)	154 (77)	36 (18)	9 (5)	
**Average number of visits per year**^(f)^				
More than 2	84 (76)	18 (16)	8 (7)	0.26
2 or less	76 (81)	16 (17)	2 (2)	
**Changed health practices**^(g)^				
Yes	85 (79)	17 (16)	5 (5)	0.96
No	75 (78)	17 (18)	4 (4)	

^a^*n* = 242: Excludes the one participant who answered “other” as their gender

^b^*n* = 238: Excludes participants who did not answer

^c^*n* = 229: Excludes participants who answered “both” and “other”

^d^*n* = 203: Excludes participants who do not have a family physician and those who did not answer

^e^*n* = 219: Excludes participants who did not answer

^f^*n* = 204: Excludes participants who did not answer

^g^*n* = 203: Excludes participants who answered “I don’t know” and those who did not answer

### FISSA’s perceptions of physicians’ competency in the treatment of malaria using HCP index score

Knowing how to quickly recognize malaria symptoms, hospitalizing children with fever higher than 39 °C, and asking patients with fever whether they travelled to tropical regions are considered as satisfactory knowledge/practices to clinically deal with malaria in non-endemic regions [27]. The confidence of FISSA in Alberta physicians’ ability to treat malaria appears to be low, with only 39% (148/382) receiving a positive score on the generated HCP index. Unfortunately, participants with low HCP index scores were less likely to indicate that they would visit a hospital if sick after a trip to an endemic region (*p* = 0.016, Table 6). Additionally, this group of FISSA was significantly more likely to believe that doctors would fail to ask for their travel history if they or their family member were sick and presented to the hospital compared to FISSA with a positive HCP score (27% vs 8% respectively, *p* = 0.002). Thirty-one percent of respondents gave neutral responses to the HCP index related questions resulting in neutral scores (Supplementary file 1). Further details are shared in Supplementary file 1.

**Table 6:** Participants’ HCP index score and malaria-related healthcare statements (*n* = 382)

Parameter	Healthcare Competency Perception (HCP) Score			
	Low (< 5)	Neutral (5)	High (> 5)	*P* value
**Would seek Canadian medical advice before travelling**^(a)^				
Yes	56 (32)	46 (26)	75 (42)	0.07
No	51 (28)	67 (37)	62 (34)	
**Listed healthcare personnel as one of their sources for information about malaria**				
Yes	71 (31)	65 (28)	96 (41)	0.27
No	45 (30)	53 (35)	52 (35)	
**If you or a family member were sick after a trip, you would:**^(b)(c)^				
Go to the hospital/family doctor				
Yes	80 (29)	82 (30)	113 (41)	***p* < 0.05**
No	12 (32)	18 (49)	7 (19)	
Treat at home/traditional recourse				
Yes	19 (39)	14 (29)	16 (33)	0.33
No	73 (28)	86 (33)	104 (40)	
**If you or a family member had a fever after a trip, you would:**^(b)(d)^				
Go to the hospital/family doctor				
Yes	84 (31)	82 (30)	106 (39)	0.42
No	10 (23)	17 (40)	16 (37)	
Treat at home/traditional recourse				
Yes	14 (29)	18 (38)	16 (33)	0.60
No	80 (30)	81 (30)	106 (40)	
**You would mention recently having travelled to an endemic region if you or your child were sick and admitted to the hospital**^(e)^				
Yes	89 (29)	90 (30)	126 (41)	0.13
No	14 (38)	14 (38)	9 (24)	
**If you or your child were sick and admitted to the hospital, doctors would inquire about your recent travel history**^(f)^				
Yes	59 (27)	61 (28)	100 (45)	***p* < 0.01**
No	22 (51)	12 (28)	9 (21)	

^a^Includes advice from Canadian doctors, pharmacists & travel clinics

^b^ = multiple answers possible, some participants would do both

^c^*n* = 312: 70 participants did not answer

^d^*n* = 315: 67 participants did not answer

^e^*n* = 342: 38 participants answered “I don’t know” and 2 did not answer

^f^*n* = 263: 117 participants answered “I don’t know” and 2 did not answer

## Discussion

FISSA play a critical role in the Edmonton’s Francophone community. As a culturally and linguistically diverse population, FISSA present interesting health particularities. We explored FISSA’s health attitudes and practices as well as experiences in accessing and navigating health services in Edmonton, a predominantly English-speaking city. Like other studies [11, 12], we demonstrated that finding a French speaking health professional remains a challenge in language minority settings, despite French being one of Canada’s official languages. We also demonstrated that biomedicine was the preferred type of care among most FISSAs and that those who preferred biomedicine were more likely to consult a doctor or present to the emergency department if sick after traveling. Those who indicated preferring traditional care, however, were more likely to stay home or to seek alternatives. These findings are in accordance with data from a study [4] that underscored treatment preferences as potential barriers to accessing healthcare services for immigrants in Canada.

Immigrants’ ability to have health insurance differed according to social class. Employment and financial resources increase the likeliness of having health insurance plans with full coverage [28]. We observed that first-generation FISSA who had lived in Canada for shorter periods of time (less than 3 years) were less likely to have coverage. While health insurance is more often quoted as a barrier to care for undocumented immigrants [28] participants in this study were all documented FISSA who have lived in Canada for more than a year. The reasons for which many FISSA reported not having health insurance should therefore be examined in future studies.

In general, immigrants’ healthcare practices differed with length of stay in host country [29] and outcomes of healthcare experiences [4, 10] or health insurance [28]. In this study, we observed that although 38% of FISSA indicated having changed their health practices through various practices such as an increase in checkups and improvements to sanitary practices, there was still a considerable number of participants (43%) who indicated having made no changes. A common justification for this was simply that they felt no need to do so (52%). There were no associations between having changed practices and sociodemographic characteristics nor years since arrival to Canada. These data suggest that FISSA probably rely on other resources for healthcare practices. This hypothesis is in line with findings that showed that immigrants may rely on traditional medicine [30, 31]. Since access to basic health services without cost is available for all residents regardless of income, employment or health status in Canada, further studies are needed to characterize the reasons why a high proportion of FISSA preferred to keep their general healthcare practices.

Studies have pointed out that language is the dominant barrier as many immigrants find it difficult to understand healthcare providers when services are not available in their first language [11, 18]. In our study only 12% of participants indicated language as an important factor when looking for healthcare services. Instead, the most cited factors were healthcare provider competency (18%) and quality of care (14%). FISSA who were more comfortable speaking in French were still much more likely to consider language as an important factor than those who preferred English (*p* = 0.021). Another commonly sought out factor was proximity (12%). This could present a potential dilemma to patients who must sometimes choose between the two. Furthermore, we showed that even though 46% (95/207) of FISSA mentioned being more at ease in French and having received care in Alberta from English-speaking doctors, a high proportion of them reported that access to medical treatment was easy. In addition, they mentioned having good comprehension during their visits and were satisfied with the care they received. Contrary to other studies [11, 12, 28], findings from this study suggest that with respect to living in French minority settings, being most at ease in French and receiving healthcare services in English rather than French surprisingly did not seem to significantly affect participants overall satisfaction with the care they received. Notwithstanding, the importance of language in FISSA access to healthcare with regards to comprehension was not completely excluded as 12% of FISSA having received care in English despite being more comfortable in French reported low comprehension during their medical visit compared to 3.7% for those who had been tended to in French. Participants in this study are highly educated with a majority (76%) of them having a post-secondary degree [20]. Furthermore, a significant proportion of FISSA earned higher than average income (data not shown). Consequently, we hypothesize that the FISSA population enrolled in this study was proficient enough in English to navigate the Albertan Health system, mitigating the most noticeable impacts of not receiving French care. Further research should be considered using variables including levels of education and bilingualism to provide generalizable knowledge on access to healthcare for FISSA.

Although FISSA were satisfied with the care they received in Alberta, confidence in the healthcare system’s ability to effectively treat malaria remained low. Only 39% (148/382) of FISSA received a positive score on the HCP index. This is consistent with articles that reported knowledge gaps among Canadian health professionals in the diagnosis and management of patients with tropical diseases including malaria [1, 23]. Based on the assumption that Francophones living in French minority settings, unaddressed language barriers can lead to a lack of confidence in the quality of care received [11], we consider that further studies are required to better address the origins of the decreased confidence among FISSA who were bilingual or more comfortable in English.

Overall, this study reveals interesting insight into FISSA access to healthcare services in Edmonton, a predominantly English-speaking community. Encouragingly, it shows that FISSA succeed in accessing Edmonton healthcare. However, the positive experience of FISSA with Albertan healthcare did not seem to increase their confidence in Canadian healthcare professionals’ ability to effectively manage malaria. Given that this population is particularly at risk of contracting the disease, more needs to be done to address this. Additionally, the uniqueness of FISSA multiple minority status which includes language and racial minorities [32] warrants further study, with an intersectional view to highlight their specific challenges and identify FISSA-oriented potential solutions.

Data from this study prompted some recommendations including the awareness of active offer [11] that should be considered to better characterize a unique population like FISSA. Active offer has been promoted in French professional training programs in Alberta. However, significant efforts are still required to promote greater general awareness of active offer in the FISSA population. More importantly, FISSA should be aware that receiving healthcare in the language in which they are most comfortable is already an important step for a successful diagnosis, treatment, and the quality of care in general. Creating an efficient partnership with organizations that usually provide services (education, health, settlement etc.) to FISSA, Francophone African health professionals, Francophone African associations, community leaders as well as ethnic media would help to initiate a culturally sensitive educational awareness program within this community. Furthermore, with the discontinuation of in-person interpretation services in Alberta and the well-documented lack of confidence in translators and interpreters [28, 33], increasing the visibility of active offer in healthcare locations is required.

A major limitation of this study is the fact that the survey format might have probably selected for a more educated subgroup of FISSAs that is less susceptible to barriers to care. Likewise, we have not considered all health delivery system characteristics including FISSA’s knowledge and familiarity with the Albertan healthcare system, the types of services FISSA had access to and the procedures for patient care to evaluate FISSA’s access to healthcare and satisfaction. Another important limitation is the fact that ease of access was only assessed for participants who had managed to receive care in the first place. Unfortunately, this may mean that FISSA who failed to access care due to significant barriers would not have been properly highlighted within the study.

## Conclusions

Our study provides an important insight into FISSA experience with Alberta’s healthcare system. It is the first to characterize FISSA health-related views and experiences with healthcare in Edmonton.

Despite reporting a relative ease in access to care and high satisfaction levels, many Edmonton FISSA still do not have French-speaking physician. FISSA confidence in Canadian doctors to effectively identify and manage malaria also remains low, decreasing their likeliness to seek medical help if sick after returning from SSA.

Given the well-documented detriments to diagnosis, treatment and overall patient care that pose language barriers, it is concerning that many FISSA did not consider language and communication as important factors when looking for care. While active offer in French has been promoted by professional programs in Alberta, community-level initiatives aiming to increase patient awareness of its benefits should be acknowledged and supported. Health professionals need to play an active role in malaria prevention, inquiring about travel plans and offering reliable information would work to increase FISSA confidence.

### Supplementary Information

**Additional file 1:** Latent factor analysis: Participants’ Healthcare Competency Perception (HCP). **Supplementary Table 1S.** Factor analysis for participants’ Healthcare Competency Perception (382 participants). **Supplementary Figure 1S.** Participants’ HCP Score Distribution.

## Data Availability

The datasets used and/or analysed during the current study are available from the corresponding author on reasonable request.

## Abbreviations

FISSA: Francophone Immigrants from Sub-Saharan Africa
SSA: Sub-Saharan Africa
HCP: Healthcare Competency Perception
