# Supplementary material for: Access to healthcare services and confidence in healthcare professionals’ management of malaria: the views of Francophone sub-Saharan African Immigrants living in western Canada
Source: BMC Public Health. 2023 Dec 8;23:2456. doi: 10.1186/s12889-023-17266-3 (PMC10704657; doi:10.1186/s12889-023-17266-3)
Supplement: Supplementary file 1 — Additional file 1: Latent factor analysis: Participants’ Healthcare Competency Perception (HCP). Supplementary Table 1S. Factor analysis for participants’ Healthcare Competency Perception (382 participants). Supplementary Figure 1S. Participants’ HCP Score Distribution. [file 12889_2023_17266_MOESM1_ESM.docx]

**Supplemental file 1: Latent factor analysis: Participants’ Healthcare competency perception (HCP)**

Three questionnaire items (Part V) dealt with participants' perception of Canadian doctors’ competency in the treatment of malaria.

Q18. Do you agree with the following statement: "Doctors in Canada do not know how to recognize malaria”?

Q20. Do you agree with the following statement: "Doctors in Canada tend to hospitalize children with malaria when it is not necessary"?

Q21. Do you agree with the following statement: "Doctors in Canada tend to do a lot of testing for children with malaria when it is not often necessary"?

The answers to these questions were on a range of strongly disagree – disagree- neutral – agree - strongly agree.

We recorded factor levels for each of these questions to shape a similar direction for the responses to all items by assigning numerical values. Henceforth, the coding was: “strongly disagree” =1, “disagree” = 2, “neutral” = 3, “agree” = 4 and “strongly disagree” = 5.

We then included all three items in a latent factor analysis (R, function *factanal*) with a single factor to measure the HCP competency perception.

The results of the factor analysis are shown below

**Supplementary Table 1S. Factor analysis for participants’ healthcare competency perception (382 participants)**

| **Question** | **Uniqueness** | **Factor loading** |
| --- | --- | --- |
| Q18 | 0.903 | 0.312 |
| Q20 | 0.549 | 0.672 |
| Q21 | 0.131 | 0.932 |

Scores on the latent factor were generated as an index of healthcare competency perception (HCP), given by the histogram (Supplementary Figure 1S) with low to high competency perception going from lowest to highest score.

Scores positively correlated with likelihood of hospital visit if sick after a trip to an endemic region, as well as likelihood to be asked about recent travel history if admitted to the hospital.

**Supplementary Figure 1S. Participants’ HCP Score Distribution**


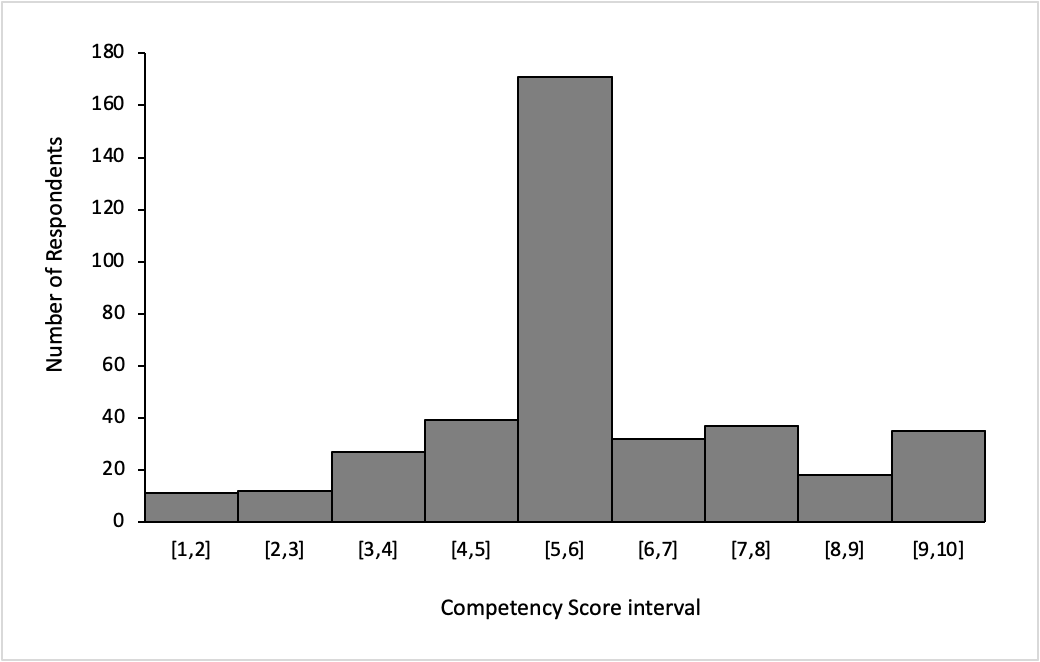


Reference

1. *Posit*. (n.d.). Posit. Retrieved November 28, 2022, from https://www.posit.co/.
